# Supplementary material for: Benchmarking of variant pathogenicity prediction methods using a population genetics approach
Source: Bioinform Adv. 2025 Sep 24;5(1):vbaf227. doi: 10.1093/bioadv/vbaf227 (PMC12579982; doi:10.1093/bioadv/vbaf227)
Supplement: vbaf227_Supplementary_Data [file vbaf227_supplementary_data.pdf]

Supplemental Note 1

"Chaining" of filters refers to the process of incremental addition of quality control (QC) filters applied to a variant set. For example, filter F_1 can be "REVEL>0.75". Naturally, any two chains made of the same QC filters will produce identical variant sets, regardless of the order in which these filters are applied in each of the two chains.

To test all possible combinations of filters $F_1, F_2, \dots, F_N$ for changes in the CAPS value, we used the following algorithm:

1. Make a list of all N filters for the analysis
2. Select maximum chain length M (i.e. the maximum number of filters to be included in a single chain, $M < N$)
3. For chain length from 1 to M:
    1. Generate all possible combinations of filters for the current length
    2. Apply each of the combinations, thus generating a new variant set
    3. Calculate CAPS on each of the new variant sets
    4. Select the maximum CAPS value for the current iteration

Hence, chains of 1 will be $(F_1), (F_2), (F_3), \dots$; chains of 2 will be $(F_1 \times F_2), (F_1 \times F_3), (F_2 \times F_3)$, ..., and so on.

Supplemental Figures

ClinGen Predictors

Source	Score Range	Score	Impact Threshold	Prediction
REVEL (meta-predictor)	0 to 1	0.782	>0.75	higher score = higher pathogenicity

Other Predictors

Source	Score Range	Score	Impact Threshold	Prediction
SIFT	--	0.005, 0.005	<0.049	D, D
PolyPhen2-HDIV	0 to 1	0.995, 0.995	--	D, D
PolyPhen2-HVAR	0 to 1	0.893, 0.893	>0.447	P, P
LRT	0 to 1	0.004033	--	N
MutationTaster	0 to 1	0.997549, 0.997549	>0.5	D, D
MutationAssessor	-0.5135 to 6.49	3.965, 3.965	>1.935	H, H
FATHMM	-16.13 to 10.64	-3.31, -3.31	<-1.51	D, D
PROVEAN	-14 to +14	-4.02, -4.02	<-2.49	D, D
MetaSVM (meta-predictor)	-2 to +3	0.9853	>0	D
MetaLR (meta-predictor)	0 to 1	0.8946	>0.5	D

Figure S1. Example of a ClinGen report for a missense variant as shown in ClinGen's Variant Curation Interface. The thresholds displayed are relevant as of 2024/09/25.

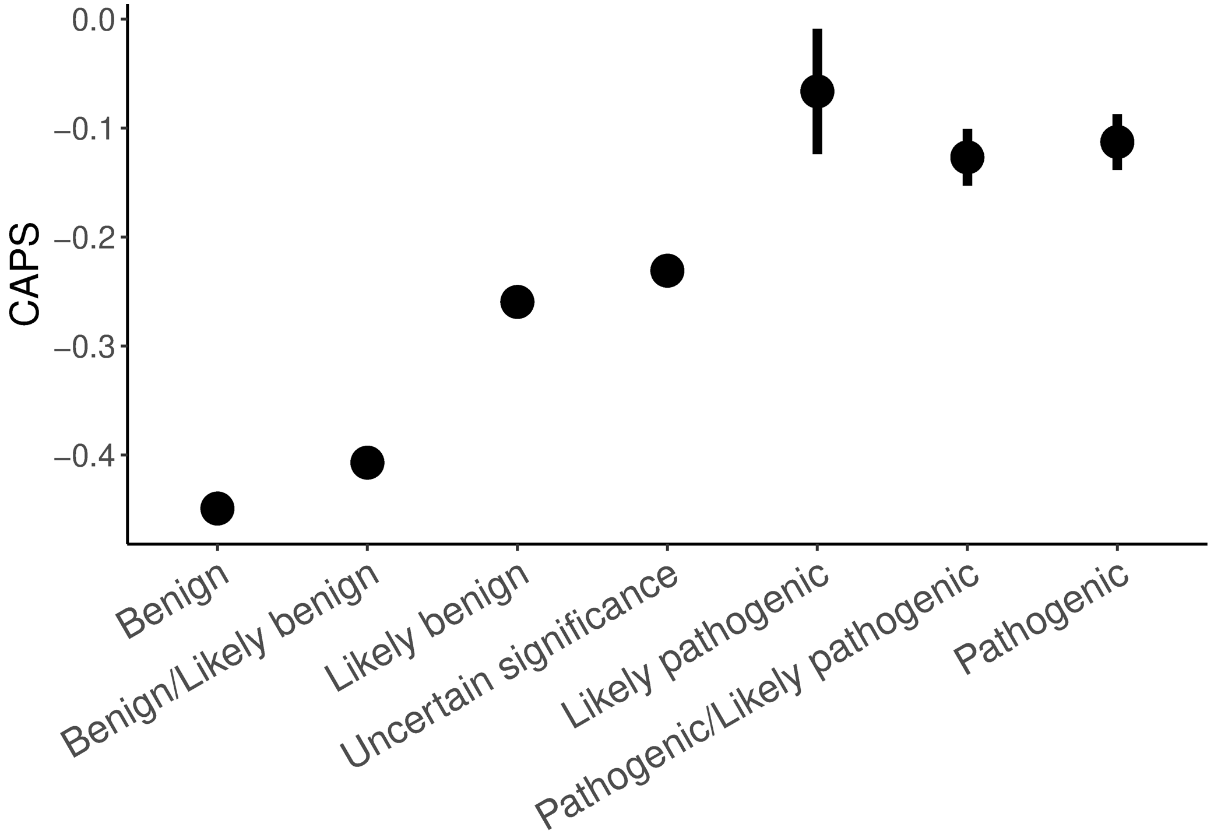

Figure S2. CAPS scores for groups of variants stratified by ClinVar pathogenicity. Only germline variants with at least two independent submitters and no conflicting interpretations were included.

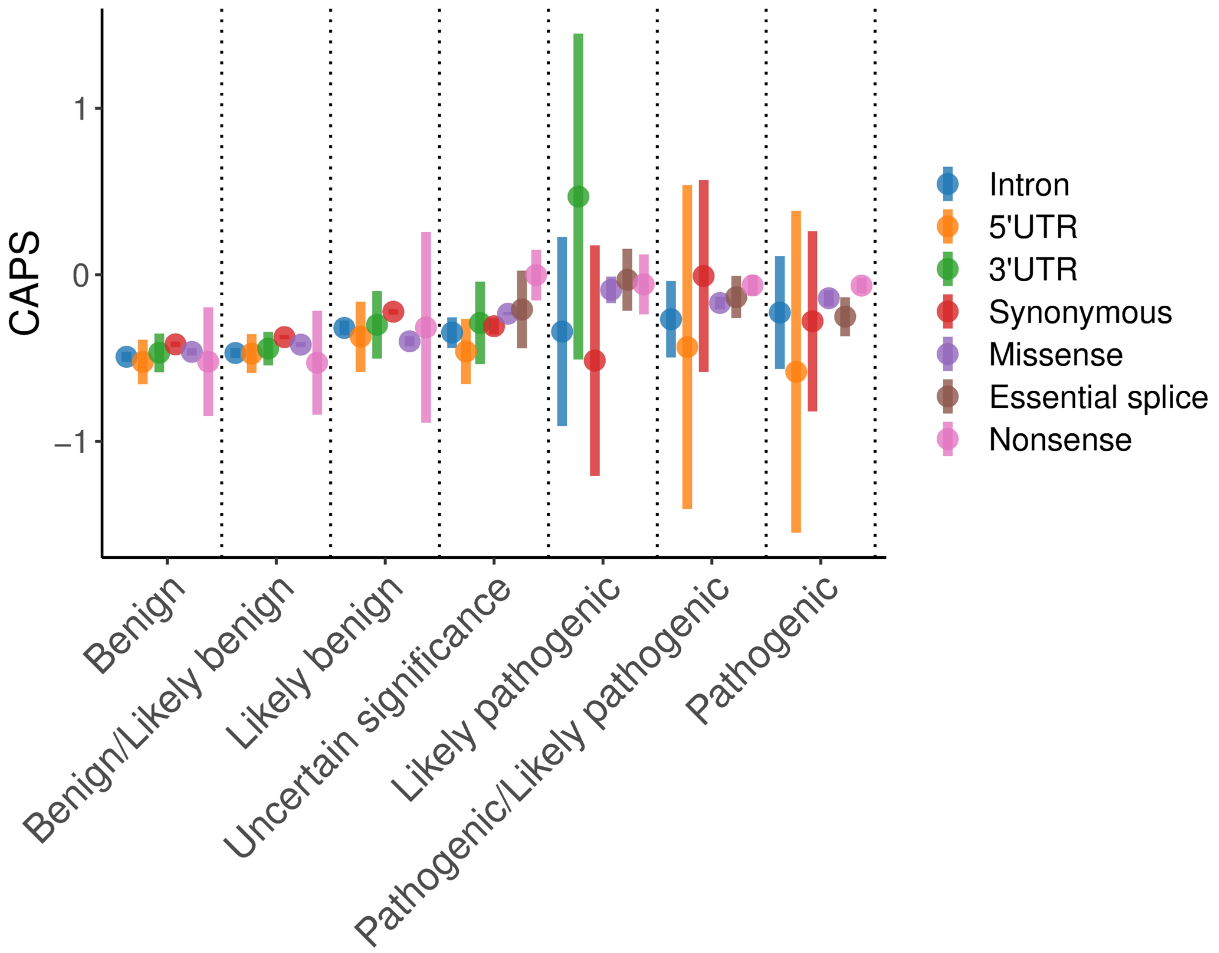

Figure S3. CAPS scores for groups of variants stratified by ClinVar pathogenicity and variant type. Only germline variants with at least two independent submitters and no conflicting interpretations were included.

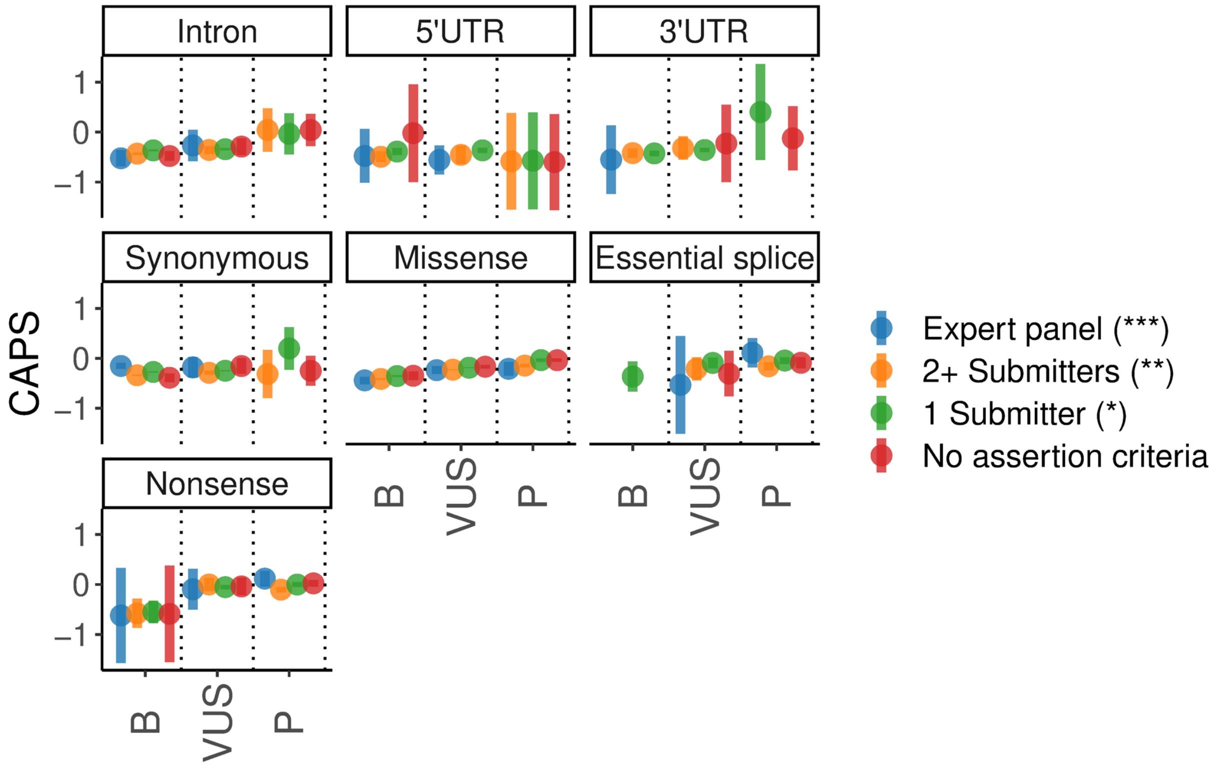

Figure S4. CAPS scores for all ClinVar variants split by review status and variant type. “Benign” (“B”) and “Pathogenic” (“P”) categories include “Likely benign” and “Likely pathogenic” categories respectively.

Supplemental Tables

Table S1. The total number of variation observed in each variant class after preprocessing.

**Class**	**Total**
Intron	2436006
5’UTR	108405
3’UTR	160419
Synonymous	2173989
Missense	4547687
Essential splice	65907
Nonsense	132974

Table S2. Change in CAPS scores during the process of finding supporting evidence for the key pathogenicity signal in a variant report, using all reference-free combinations. ClinGen report was used as an example. REVEL score of 0.118 was used as a reference. Chain length of 1 indicates one filter (based on one tool-threshold pair), chain length of 2 indicates two filters in combination, etc. Missense variants only.

**Chain length**	**Max CAPS**	**CAPS distance to reference**	**CAPS increase (percent)**
1	0.0849	0.0336	NA
2	0.0959	0.0226	32.6
3	0.0994	0.0190	15.7
4	0.102	0.0169	11.3
5	0.103	0.0158	6.19
6	0.103	0.0151	4.69
7	0.104	0.0146	3.43
8	0.104	0.0145	0.511
9 (all)	0.101	0.0172	-18.7

Table S3. Per-group statistics for ClinVar-annotated germline variants. Subsets containing all variants (ALL) and variants with at least two independent submitters and no conflicting interpretations (high-confidence, HC) are compared.

**Clinical significance**	**Total (ALL)**	**Proportion singletons (ALL)**	**Total (HC set)**	**Proportion singletons (HC set)**
Benign	17278	0.006	6409	0.000
Benign/Likely benign	11543	0.002	11508	0.002
Likely benign	40264	0.149	7083	0.141
Uncertain significance	83162	0.199	17471	0.171
Pathogenic/Likely pathogenic	1505	0.214	1442	0.212
Likely pathogenic	3252	0.366	365	0.315
Pathogenic	7708	0.329	1413	0.220

Table S4. Variant numbers for Figure 2 illustrating the effect of using stringent thresholds and "chaining" of filters on resulting CAPS scores.

**Group**	**Thresholds**	**Number of variants**
SIFT	Less stringent	3265568
CADD	Less stringent	3244444
PolyPhen	Less stringent	2722536
All 3	Less stringent	2483104
SIFT	More stringent	1858218
CADD	More stringent	2349036
PolyPhen	More stringent	1264886
All 3	More stringent	1005175

Table S5. Variant numbers for Figure 3 and Figure 5, illustrating CAPS scores for individual filters and combinations of filters.

**Group**	**Number of variants**
REVEL	240971
SIFT	1848485
PolyPhen	1691426
MutationTaster	3706267
MutationAssessor	1602851
FATHMM	669160
PROVEAN	1458721
MetaSVM	628402
MetaLR	690356
CADD	2458634
REVEL & SIFT	228382
REVEL & PolyPhen	231264
REVEL & MutationTaster	240908
REVEL & MutationAssessor	220665
REVEL & FATHMM	175599
REVEL & PROVEAN	230387
REVEL & MetaSVM	229331
REVEL & MetaLR	225766

Group	Number of variants
REVEL & CADD	240816
SIFT & PolyPhen	1308310
SIFT & MutationTaster	1846668
SIFT & MutationAssessor	1217062
SIFT & FATHMM	377341
SIFT & PROVEAN	1188928
SIFT & MetaSVM	507308
SIFT & MetaLR	507380
SIFT & CADD	1596622
PolyPhen & MutationTaster	1689684
PolyPhen & MutationAssessor	1173121
PolyPhen & FATHMM	355732
PolyPhen & PROVEAN	1115918
PolyPhen & MetaSVM	518183
PolyPhen & MetaLR	516255
PolyPhen & CADD	1583973
MutationTaster & MutationAssessor	1601038
MutationTaster & FATHMM	668408
MutationTaster & PROVEAN	1457185
MutationTaster & MetaSVM	628109

Group	Number of variants
MutationTaster & MetaLR	690037
MutationTaster & CADD	2455747
MutationAssessor & FATHMM	331150
MutationAssessor & PROVEAN	1069397
MutationAssessor & MetaSVM	477210
MutationAssessor & MetaLR	476010
MutationAssessor & CADD	1389928
FATHMM & PROVEAN	324846
FATHMM & MetaSVM	422472
FATHMM & MetaLR	500074
FATHMM & CADD	502084
PROVEAN & MetaSVM	464763
PROVEAN & MetaLR	458839
PROVEAN & CADD	1308087
MetaSVM & MetaLR	587016
MetaSVM & CADD	600186
MetaLR & CADD	617033

Table S6. Variant numbers for Figure 4 illustrating CAPS scores for ClinVar variants split by review status and for an in-house selection of CHD VUSs.

**Group**	**Review status**	**Number of variants**
CHD VUS	reviewed by VCCRI CHD expert panel	31
Pathogenic	reviewed by expert panel	205
VUS	reviewed by expert panel	340
Benign	reviewed by expert panel	757
Pathogenic	criteria provided, multiple submitters, no conflicts	2063
Benign	criteria provided, multiple submitters, no conflicts	14941
VUS	criteria provided, multiple submitters, no conflicts	22175
Pathogenic	criteria provided, single submitter	3062
Benign	criteria provided, single submitter	24652
VUS	criteria provided, single submitter	77699
Benign	no assertion criteria provided	392
VUS	no assertion criteria provided	1342
Pathogenic	no assertion criteria provided	2309
